# Predictive model for long-term weight recovery after gastrectomy for gastric cancer: an introduction to a web calculator

**DOI:** 10.1186/s12885-023-11050-7

**Published:** 2023-06-23

**Authors:** Chul-Hyo Jeon, Ki Bum Park, Sojung Kim, Ho Seok Seo, Kyo Young Song, Han Hong Lee

**Affiliations:** 1 Department of Surgery, Division of Gastrointestinal Surgery, Uijeongbu St. Mary’s Hospital, College of Medicine, The Catholic University of Korea, 271, Cheonbo-ro, Uijeongbu-si, Gyeonggi-do 11765 Republic of Korea; 2 Department of Surgery, Division of Gastrointestinal Surgery, St. Vincent’s Hospital, College of Medicine, The Catholic University of Korea, 93, Jungbu-daero, Paldal-gu, Suwon-si, Gyeonggi-do 16247 Republic of Korea; 3 Department of Surgery, Division of Gastrointestinal Surgery, Seoul St. Mary’s Hospital, College of Medicine, The Catholic University of Korea, 222, Banpo-Daero, Seocho-Gu, Seoul, 06591 Republic of Korea

**Keywords:** Stomach neoplasms, Gastrectomy, Internet, Weight gain, Nomograms

## Abstract

**Background:**

Weight changes after gastrectomy affect not only quality of life but also prognosis and survival. However, it remains challenging to predict the weight changes of individual patients. Using clinicopathological variables, we built a user-friendly tool to predict weight change after curative gastrectomy for gastric cancer.

**Methods:**

The clinical data of 984 patients who underwent curative gastrectomy between 2009 and 2013 were retrospectively reviewed and analyzed. Multivariate logistic regression was performed to identify variables predictive of postoperative weight change. A nomogram was developed and verified via bootstrap resampling.

**Results:**

Age, sex, performance status, body mass index, extent of resection, pathological stage, and postoperative weight change significantly influenced postoperative weight recovery. Postoperative levels of hemoglobin, albumin, ferritin and total iron-binding capacity were significant covariates. The nomogram performed well (concordance index = 0.637); calibration curves indicated appropriate levels of agreement. We developed an online weight prediction calculator based on the nomogram (http://gc-weightchange.com/en/front/).

**Conclusions:**

The novel, Web-calculator based on the predictive model allows surgeons to explore patient weight patterns quickly. The model identifies patients at high risk for weight loss after gastrectomy; such patients require multidisciplinary medical support.

**Supplementary Information:**

The online version contains supplementary material available at 10.1186/s12885-023-11050-7.

## Introduction

Gastric cancer remains the third-leading cause of cancer-related death worldwide (the Global Cancer Observatory estimated 783,000 deaths in 2018) despite marked declines in many countries [1]. Korea exhibits one of the highest national incidences of gastric cancer at 41.4 cases per 100,000. The prevalence of early gastric cancer (EGC) has increased since the introduction of the Korean National Health Screening Program, which includes endoscopy [2, 3]. The postoperative quality of life of gastrectomy patients requires attention. Weight and body composition changes after gastrectomy may reflect reduced physical activity and oral intake, and excessive catabolism associated with inflammatory responses to surgical stress [4]. Changes in weight and body composition adversely affect nutritional status, quality of life, and compliance with adjuvant chemotherapy, reducing survival [5]. Body weight loss greater than 10% after curative gastrectomy is associated with persistent pain, diarrhea, nausea/vomiting, and deterioration of all functional aspects of life [6]. However, few studies have identified patients at risk for excessive weight loss, or its causes. Moreover, there are few simple tools that predict weight changes with consideration of individual patient characteristics. We identified factors affecting weight change after radical gastrectomy and developed an accessible user-friendly tool based on a nomogram to predict such changes.

## Materials and methods

### Patient population and data collection

In total, 1,835 patients who underwent curative gastrectomy for gastric cancer between 2009 and 2013 at Seoul St. Mary’s Hospital were recruited. The inclusion criteria were primary gastric cancer, no other malignancy, no preoperative chemotherapy, no distant metastasis, R0 resection (no residual macroscopic or microscopic tumor), regular outpatient follow-up without disease recurrence for more than 5 years, and no missing values. Patients were excluded if their postoperative weights were not recorded, or if they were lost to follow-up or died within 5 years after surgery. Ultimately, 984 patients were enrolled. A flowchart of enrollment is shown in Supplementary Fig. 1.

Surgeons specializing in gastric cancer performed all operations based on the Korean and Japanese Guidelines for Gastric Cancer [7, 8]. We collected demographic information (age, sex, height, and weight), operative data, morbidities, tumor stages, postoperative recovery data, and recurrence and survival. We divided the extent of surgery into total gastrectomy (TG) and subtotal gastrectomy (STG). The majority of STG groups in this study were patients who underwent distal gastrectomy. Proximal gastrectomy and pylorus-preserving gastrectomy were also classified and analyzed as STG. Preoperative clinical characteristics and postoperative complications were categorized using the Eastern Cooperative Oncology Group (ECOG) [9] and the Clavien-Dindo criteria [10], respectively. Pathological staging was based on the 8th American Joint Committee on Cancer (AJCC) TNM guidelines [11]. The histological type was categorized as differentiated or undifferentiated. Poorly differentiated tubular, signet ring cell, and mucinous adenocarcinomas were considered undifferentiated. Regular follow-up was scheduled at 3- and 6-month intervals for advanced and early gastric cancer patients, respectively, for the first 3 years and every 12 months thereafter, and included measurement of tumor-marker levels, abdominal computed tomography, and endoscopy. The observation period was the time from the date of surgery to the time of death or loss to follow-up, whichever occurred first. Overall survival (OS) was calculated from the date of primary gastrectomy to the date of death from any cause or the time of the last follow-up.

### Primary factors affecting weight change after gastrectomy

#### Body weight identification, measurement and classification

**Fig. 1:**
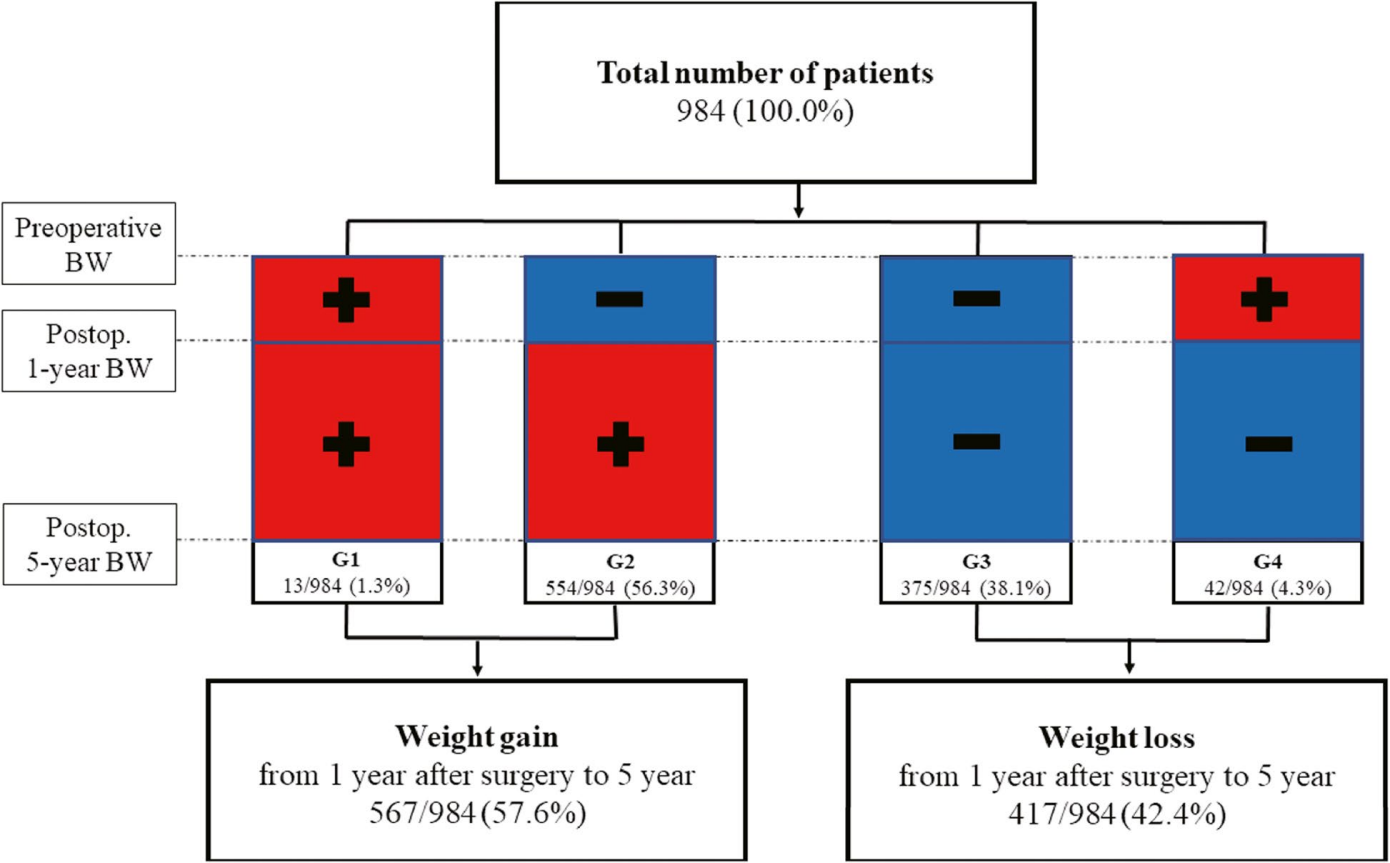
Body weights (BWs) before surgery and at 1 year and 5 years postoperatively were compared. G1 indicates patients exhibiting continuous weight gain after surgery; G2 are those whose weight increased from 1 year after surgery (after weight loss); G3 includes patients who consistently lost weight after surgery; and G4 represents those who lost weight from 1 year after surgery (after weight gain). A red square with a plus sign indicates an increase in weight over time, and a blue square with a minus sign indicates weight loss

Previous studies have reported that only slight weight reduction occurs after the first 6 months after gastrectomy, regardless of the surgical method; the maximum weight change becomes evident at 12 months after surgery and then either stabilizes or improves over time [12, 13]. However, in one study, weight loss of more than 10% was maintained even 2 years after operation in more than half of all patients [6]. Thus, the importance of initial weight change has been universally accepted. We hypothesized that patients’ initial weight change would predict their long-term weight change. We viewed the weight change at 1 year after surgery as a significant parameter; we collected patients’ weights before surgery and at 1 and 5 years after surgery. A weight change of less than 2.5% was considered insignificant. Patients were classified by weight change over time, and prognostic factors according to each group were sought. The groups were as follows (Fig. 1):

- G1: Continuous weight gain after surgery
- G2: After weight loss, an increase in weight from 1 year after surgery
- G3: Continuous weight loss after surgery
- G4: After weight gain, a decrease in weight from 1 year after surgery

### Blood analyses

We assessed the potential correlations between weight change and blood status; we collected the preoperative and 1-year postoperative hemoglobin (Hb) levels, the mean corpuscular volume (MCV), the mean corpuscular hemoglobin (MCH) level, the mean cell hemoglobin concentration (MCHC), the total iron-binding capacity (TIBC), and the levels of albumin, iron, transferrin, folate, ferritin, and vitamin B-12. TIBC and ferritin status were classified as low, normal, or high using in-house reference values because normal range of these values differs slightly by institution.

### Statistical analysis

Continuous values are presented as means with standard deviations and were compared using the Student t-test; the chi-square or Fisher exact test (as appropriate) was employed to compare categorical variables. Cox’s proportional hazard regression models were used to calculate hazard ratios (HRs) with 95% confidence intervals (CIs). Binary logistic regression was used to identify factors that significantly influenced weight change. All variables with *P*-values < 0.10 in univariate analysis were entered into multivariate analysis to identify independent risk factors; Along with parameters with *p*-values < 0.10, variables identified as factors influencing weight change in previous studies or that the authors of this study empirically judged to affect weight change were also entered into the final model.

All analyses were performed using SPSS ver. 24.0 for Windows (IBM Corp., Armonk, NY, USA). *P* < 0.05 was considered statistically significant.

### Conventional and Web-based nomogram development

All identified independent risk factors were incorporated into a nomogram. Every variable was scored between 0–100 by drawing vertical lines and the scores were then summed. The model was validated using 1,000 bootstrap resamplings of the developmental dataset. The prognostic performance was evaluated based on the concordance index (C-index; range 0–1) with the 95% CI, and the area under the curve (AUC) of the receiver operator characteristic (ROC) plot. All data management and statistical analyses were performed using R ver. 3.5.1 (R Foundation for Statistical Computing, Vienna, Austria). We prepared a Web-based version of the nomogram that automatically calculates the final score when the parameters are input.

## Results

### Clinicopathological characteristics

Supplement Table 1 presents the general clinicopathological characteristics of the 984 patients. The median follow-up was 73 ± 17.33 months. Of all patients, 278 were aged 65 years or older (28.3%); males accounted for 66.3% (*n* = 652). Most patients were of ECOG grade 0 or 1 (*n* = 969, 98.5%). Of all patients, 37.5% had a body mass index (BMI) < 23 kg/m^2^ before surgery; 75.4% of patients underwent subtotal gastrectomy. Early gastric cancer patients constituted 78.8% of the total cohort.

**Table 1:** Patient characteristics according to weight change from 1 to 5 years after gastrectomy

Variables, n(%)	Weight gain (*N* = 567, 100%)	Weight loss (*N* = 417, 100%)	*p*-value
**Age (years)**			**< 0.001**
< 65	436 (76.9%)	270 (64.7%)	
≥ 65	131 (23.1%)	147 (35.3%)	
**Sex**			**0.001**
Male	353 (62.3%)	299 (71.7%)	
Female	214 (37.7%)	118 (28.3%)	
**ECOG**			**0.055**
0–1	562 (99.1%)	407 (97.6%)	
≥ 2	5 (0.9%)	10 (2.4%)	
**Preoperative BMI (kg/m**^**2**^**)**			0.960
< 23	213 (37.6%)	156 (37.4%)	
≥ 23	354 (62.4%)	261 (62.6%)	
**Resection**			0.934
STG	427 (75.3%)	315 (75.5%)	
TG	140 (24.7%)	102 (24.5%)	
**pTNM stage**			**0.068**
EGC	435 (76.7%)	340 (81.5%)	
AGC	132 (23.3%)	77 (18.5%)	
**Postoperative weight change over 1 year**			**< 0.001**
(-) ↓	554 (97.7%)	375 (89.9%)	
( +) ↑	13 (2.3%)	42 (10.1%)	
**Hemoglobin change**			**< 0.001**
(-) ↓	95 (16.8%)	107 (25.7%)	
( +) ↑	472 (83.2%)	310 (74.3%)	
**Albumin change**			**0.067**
(-) ↓	179 (31.6%)	155 (37.2%)	
( +) ↑	388 (68.4%)	262 (62.8%)	
**TIBC class**			0.996
Low/Normal	548 (96.6%)	403 (96.6%)	
High	19 (3.4%)	14 (3.4%)	
**Ferritin class**			1.000
High	6 (1.1%)	4 (1.0%)	
Low/Normal	561 (98.9%)	413 (99.0%)	

The chi-square test was used to evaluate between-group differences in categorical variables

*Abbreviations*: *ECOG* Eastern Cooperative Oncology Group performance status, *BMI* Body mass index, *STG* Subtotal gastrectomy, *TG* Total gastrectomy, *EGC* early gastric cancer, *AGC* Advanced gastric cancer, *TIBC* Total iron-binding capacity

### Factors affecting weight change after gastrectomy

Table 1 compares the characteristics of the weight-gain and weight-loss groups at 5 years postoperatively. The percentage of patients who evidenced recovered or increased weight at 5 years (compared to at 1 year) after surgery was 57.6% (567/984). The percentages of relatively young and female patients, and those of better preoperative performance status and with more advanced disease, tended to be higher among such patients than others. Higher hemoglobin and albumin levels at 1 year after surgery predicted long-term weight gain. Patients who lost weight in the first year after gastrectomy gained significant weight during the next 4 years. MCV, MCH, MCHC, iron, transferrin, folate, and vitamin B-12 levels did not differ between the weight-gain and weight-loss groups (data not shown).

### Weight change patterns

We analyzed the weight-change patterns of the four groups. The G1 group included the highest proportions of young patients, females, and patients with lower BMI values before surgery. G2 was similar, but the mean preoperative BMI was higher. G3 contained a more significant proportion of older male patients with higher BMI values before surgery but with disease of lower severity. G4 contained the highest proportion of old males with severe disease. G4 patients had lower preoperative BMI values but the highest proportion of subtotal gastrectomy (Supplement Table 2). Almost 94% (929/984) of patients (G2 and G3) lost weight during the first year after surgery (Fig. 1).

**Table 2:** Factors predictive of weight gain during 5 years after gastrectomy

Variables, n(%)	Odds ratio	95% CI	*p*-value
**Age (years)**			**< 0.001**
≥ 65	Ref		
< 65	1.751	1.312–2.339	
**Sex**			**0.002**
Male	Ref		
Female	1.555	1.173–2.060	
**ECOG**			0.344
≥ 2	Ref		
0–1	1.735	0.554–5.436	
**Preoperative BMI (kg/m**^**2**^**)**			0.358
≥ 23	Ref		
< 23	1.140	0.862–1.509	
**Resection**			0.629
TG	Ref		
STG	1.079	0.793–1.469	
**pTNM stage**			**0.008**
EGC	Ref		
AGC	1.564	1.123–2.178	
**Postoperative weight change over 1 year**			**< 0.001**
( +) ↑	Ref		
(-) ↓	4.758	2.478–9.138	
**Hemoglobin change**			**< 0.001**
(-) ↓	Ref		
( +) ↑	1.572	1.138–2.171	
**Albumin change**			0.311
(-) ↓	Ref		
( +) ↑	1.164	0.868–1.562	
**TIBC class**			0.996
High	Ref		
Low/Normal	1.100	0.522–2.320	
**Ferritin class**			0.605
Low/Normal	Ref		
High	1.426	0.371–5.480	

*Abbreviations*: *ECOG* Eastern Cooperative Oncology Group performance status, *BMI* Body mass index, *STG* Subtotal gastrectomy, *TG* Total gastrectomy, *EGC* Early gastric cancer, *AGC* Advanced gastric cancer, *TIBC* Total iron-binding capacity

### Model predicting long-term weight change after gastrectomy

Table 2 lists the selected variables with odds ratios. Several factors were associated with long-term weight recovery: younger age, female sex, lower ECOG score (0–1), lower BMI before surgery (BMI < 23 kg/m2), subtotal gastrectomy, more advanced cancer stage, weight loss at 1 year after surgery, maintained or increased Hb and albumin levels at 1 year after surgery, high ferritin level, and low/normal TIBC.

**Fig. 2:**
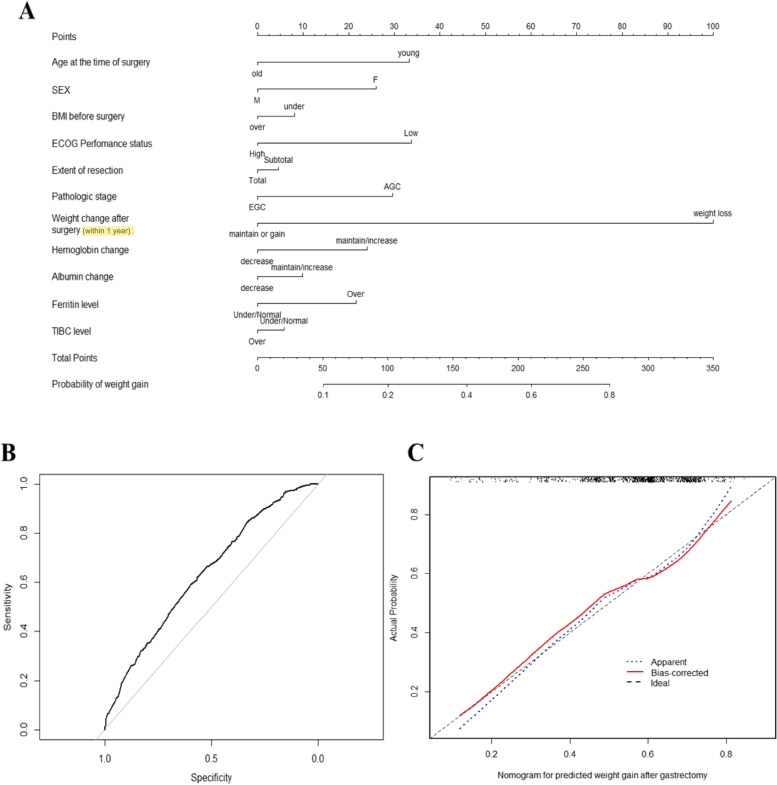
**A** Nomogram predicting the weight-gain probability at 1–5 years after gastrectomy. **B** Validation of predictive performance. Internal validation yielded a C-index of 0.637 (95% CI: 0.602–0.672). **C** The internal calibration curve revealed good agreement between nomogram predictions and actual observations; the predictive probability was 0–0.80)

**Fig. 3:**
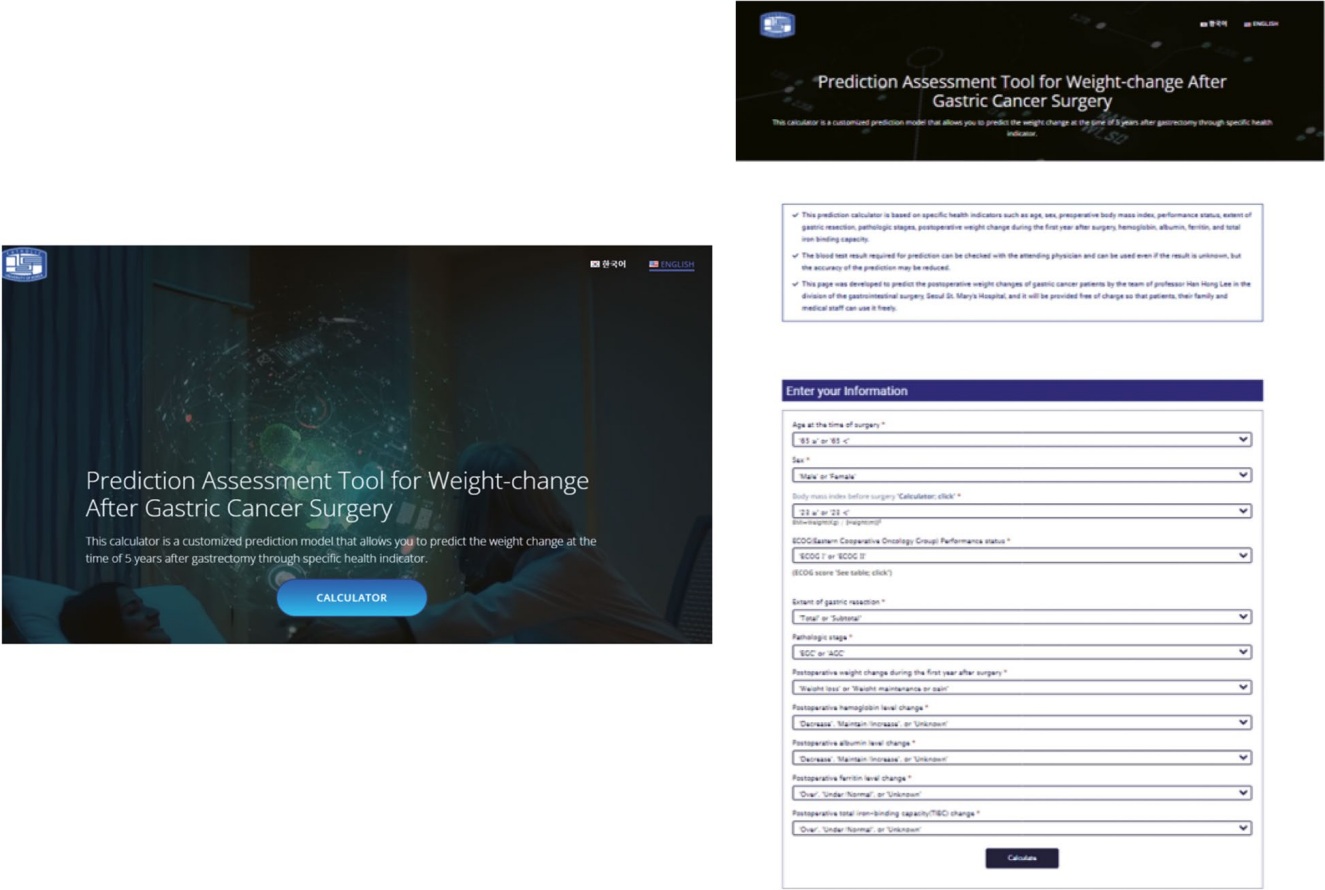
The Web-based platform for long-term weight-change prediction. See http://gc-weightchange.com/en/front/

**Fig. 4:**
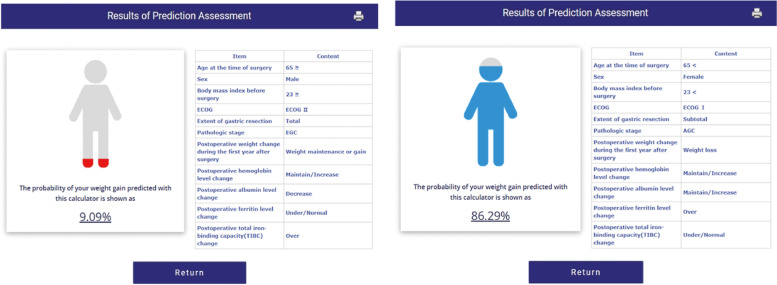
Examples of Web-based predictions. The range of values was 9.09–86.29%

Figure 2A shows the nomogram predicting the probability of weight gain at 1–5 years after surgery. The nomogram assigns a probability by summing the scores for each variable. The total score (on the bottom) indicates the probability of weight gain at 5 years after surgery. Internal validation yielded a C-index of 0.637 (95% CI 0.602–0.672) identical to the AUC of the ROC plot. The internal calibration curves revealed good agreement between the nomogram predictions and actual observations over a predictive probability range of 0–0.80 (Figs. 2B, C). Our novel web-calculator based on the nomogram is available online at http://gc-weightchange.com/en/front/ (Fig. 3). It rates the probability of weight gain at 5 years after gastrectomy from 9.09% to 86.29%. It is free and there is no need to log in (Fig. 4).

## Discussion

After curative gastrectomy for gastric cancer, most patients experience weight loss of about 10–20%; after passing a nadir, weight stabilizes or increases at about 12 months after surgery [13, 14]. Weight loss is attributable to reduced food retention in the stomach, and poor intestinal digestion caused by upper gastrointestinal tract dysfunction; this triggers post‐gastrectomy syndrome and malnutrition [15]. Consistent with previous studies, we found that the average weight loss was about 10% at 1 year after surgery (data not shown). Only 1% of patients experienced continuous postoperative weight gain. Except for such cases, most of the weight change in the first year after surgery was confirmed to be a decrease.

Weight loss after gastrectomy negatively affects quality of life and is associated with dismal survival rates [6, 16]. It is essential to identify patients who are expected to lose weight continuously or to experience difficulty in weight recovery; appropriate clinical intervention can improve their quality of life and prognosis. We are the first to construct a nomogram to predict weight change; we measured the weights of a large retrospective cohort before and after surgery. This nomogram elucidates long-term weight change after surgery, individualizes the weight-gain probability, and facilitates personalized medicine. Again, the model is fast, free, and online.

We found that age, sex, BMI, ECOG performance status, the extent of resection, disease severity, and weight change at 1 year after surgery predicted weight recovery 5 years after surgery. Changes in the levels of Hb, albumin, and ferritin, and the TIBC, also reflected weight changes after gastrectomy. In developing the weight recovery prediction model, we included factors that were not statistically significant due to the limited enrolled sample size in our study. However, based on previous research and our experience, we believed that performance status, preoperative body mass index, the extent of resection, and the degree of anemia would play significant roles in postoperative weight changes after gastrectomy [17–20]. Therefore, we incorporated these factors into our prediction model and web calculator.

Our study was conducted at an accredited institution with a high-volume gastric cancer center, which has established a well-structured database and implemented a robust long-term follow-up system. Such resources within our institution ensure the accuracy and integrity of the data utilized in this study. We could perform bootstrap resampling of the developmental data with a comprehensive and reliable database. Moreover, internal calibration curves were employed to evaluate the agreement between the nomogram predictions and the actual observations (Fig. 2B,C). The significant findings obtained through this analysis further validate the reliability and consistency of our results.

Elderly patients with gastric cancer lose more lean body mass than do others [4]. Older age and total gastrectomy are independent risk factors for post-gastrectomy malnutrition [21]; our findings are consistent with this notion. Patients who underwent total gastrectomy with a higher baseline BMI lost significantly more weight than those of subtotal gastrectomy with lower baseline BMI [13, 22, 23]. We found that subtotal gastrectomy patients evidenced better weight recovery than total gastrectomy patients, perhaps because of better calorie intake, less functional impairment, and a lower blood ghrelin level [24–27].

In this study, most patients belonging to Group 2 and Group 3 demonstrated weight loss during the first year after surgery, while Group 1, which showed steady weight gain after surgery, accounted for only a minimal percentage of all patients (1.4%). This finding is consistent with a previous study that analyzed weight change patterns in 376 patients who underwent gastrectomy, highlighting the occurrence of maximum weight change up to 12 months postoperatively [13]. The congruence between the results of our study and the previous findings suggests that patients who experience weight loss within the first year after surgery are more likely to exhibit weight recovery starting from 1 year postoperatively. Therefore, weight loss 1 year after surgery is a positive predictor for weight recovery 5 years after surgery.

We found a more significant relationship between male (compared to female) sex and post-gastrectomy body weight loss after subtotal gastrectomy; males have been reported to be more susceptible than females to lean-body-mass loss after gastrectomy [15, 28]. Consistent with previous studies, we found that females exhibited a higher probability of weight recovery than did males. Any relationship between sex and weight recovery after gastrectomy is not fully established. However, we hypothesize that the reasons for lower weight loss after gastrectomy in women are that on average women are lighter than men and have a lower BMI, and show better treatment compliance [29].

Advanced cancer is associated with preoperative cachexia, aggressive tumor behavior, and an increased tumor burden [30]. Cachexia is more negatively prognostic than is simple sarcopenia after radical gastrectomy to treat advanced gastric cancer [31]. When malignant lesions are radically resected, the tumor burden is eliminated, and the probability of weight recovery is thus expected to be high. As we excluded cases with recurrence during follow-up, we suggest that recovery from cachexia was successful.

Albumin is a prognostic tumor marker that has been of great interest; its level is closely related to nutritional and inflammatory status. The albumin level is a component of the prognostic nutritional index (PNI), the controlling nutritional status (CONUT) score, and the nutritional risk index (NRI) commonly used for nutritional evaluation after gastrectomy [21, 32, 33]. Albumin levels tend to recover for up to 5 years after surgery, reflecting weight recovery [34]. Therefore, if the albumin level at 1 year after surgery is maintained or increased, nutritional status is improving, and the probability of weight recovery is increased.

Various types of anemia can occur after gastrectomy; most are attributable to iron deficiency, abnormal vitamin B12 metabolism, or a combination thereof [23]. The most efficient test of iron deficiency is the serum ferritin assay; the total iron-binding capacity (TIBC) is prognostic of such deficiency [35, 36]. We measured the Hb and ferritin levels, and the TIBC; the latter two parameters are major components of our weight-change-prediction model.

Previous studies have indicated a significant relationship between changes in weight and body composition and compliance with adjuvant chemotherapy [5]. In our study, approximately 21% of the patients met the indication for adjuvant chemotherapy, with the majority being diagnosed with EGC. Also, for long-term survivors (> 5 years) after surgery, factors other than the administration of adjuvant chemotherapy were considered more influential, as mentioned earlier. Consequently, chemotherapy was not included as a factor in the prediction model of this study. It is important to recognize that additional potential predictors, such as lifestyle and behavioral factors, as well as individual medical conditions like diabetes mellitus and postoperative complications, could impact body weight changes after gastrectomy for gastric cancer [37]. However, considering the heterogeneity of medical conditions and the risk of incorporating too many variables into the prediction model, it may not be appropriate to include an extensive range of factors. In light of this consideration, the authors of this study carefully selected and applied clinicopathological factors that have been evaluated through various methods, aiming to create a simple yet powerful model with improved predictive ability. Notably, we are the first to construct a nomogram specifically designed to predict weight change after gastrectomy for gastric cancer.

The nomogram-calculated probability of weight gain ranged from 10% to about 80%. It is important that clinicians seek factors that increase the probability of weight gain (excluding unmodifiable clinicopathological factors) during outpatient follow-up. We developed a freely available Web-based version of our weight-prediction method; the results are immediate. Non-medical personnel can obtain minimum and maximum risks when patients and caregivers lack access to laboratory findings. According to the model, if an elderly obese male who is not physically active loses weight in the first year after total gastrectomy for early gastric cancer, the possibility of weight regain is only 19.69–40.67% regardless of the blood data, which is rather low. Such result of nomogram could be an important basis that a multidisciplinary effort for weight recovery is mandatory.

Our work had a few limitations. First, the study was retrospective, performed in a single institution, and included only Asians. Western gastric cancer patients differ; many are obese, have severe disease, and undergo preoperative adjuvant therapy. Therefore, care should be taken when generalizing the results. Second, our predictive malnutrition-risk model has not been externally validated to confirm its accuracy. However, as internal validation was good, the clinical relevance of our model is clear. Seoul St. Mary’s Hospital is a high-volume gastric cancer center with a systemized database and appropriate follow-up system, the latest protocol of including not only surgery but also chemotherapy has been consistently applied, and the data of this study are reliable [38].

In conclusion, we developed and validated a nomogram to predict weight change after gastrectomy for gastric cancer. We studied the body weights and clinicopathological features of a large cohort at various times. We constructed an online weight-prediction model based on a nomogram. This will help clinicians to derive weight patterns easily and efficiently, facilitating nutritional education and medical support. Our tool identifies patients at high risk for failure to maintain appropriate weight after gastrectomy. Such patients require comprehensive support.

## Supplementary Information

**Additional file 1: Supplementary Fig. 1.** Flow chart of the study cohort with the inclusion and exclusion criteria.

**Additional file 2: Supplementary Table 1.** Demographic and clinicopathological variables. **Supplementary Table 2.** Characteristics of the subgroups by the trends in body weight change after gastrectomy.

## Data Availability

The datasets used and/or analysed during the current study available from the corresponding author on reasonable request.
