# Supplementary figures and images for: Predictive model for long-term weight recovery after gastrectomy for gastric cancer: an introduction to a web calculator
Source: BMC Cancer. 2023 Jun 23;23:580. doi: 10.1186/s12885-023-11050-7 (PMC10288751; doi:10.1186/s12885-023-11050-7)

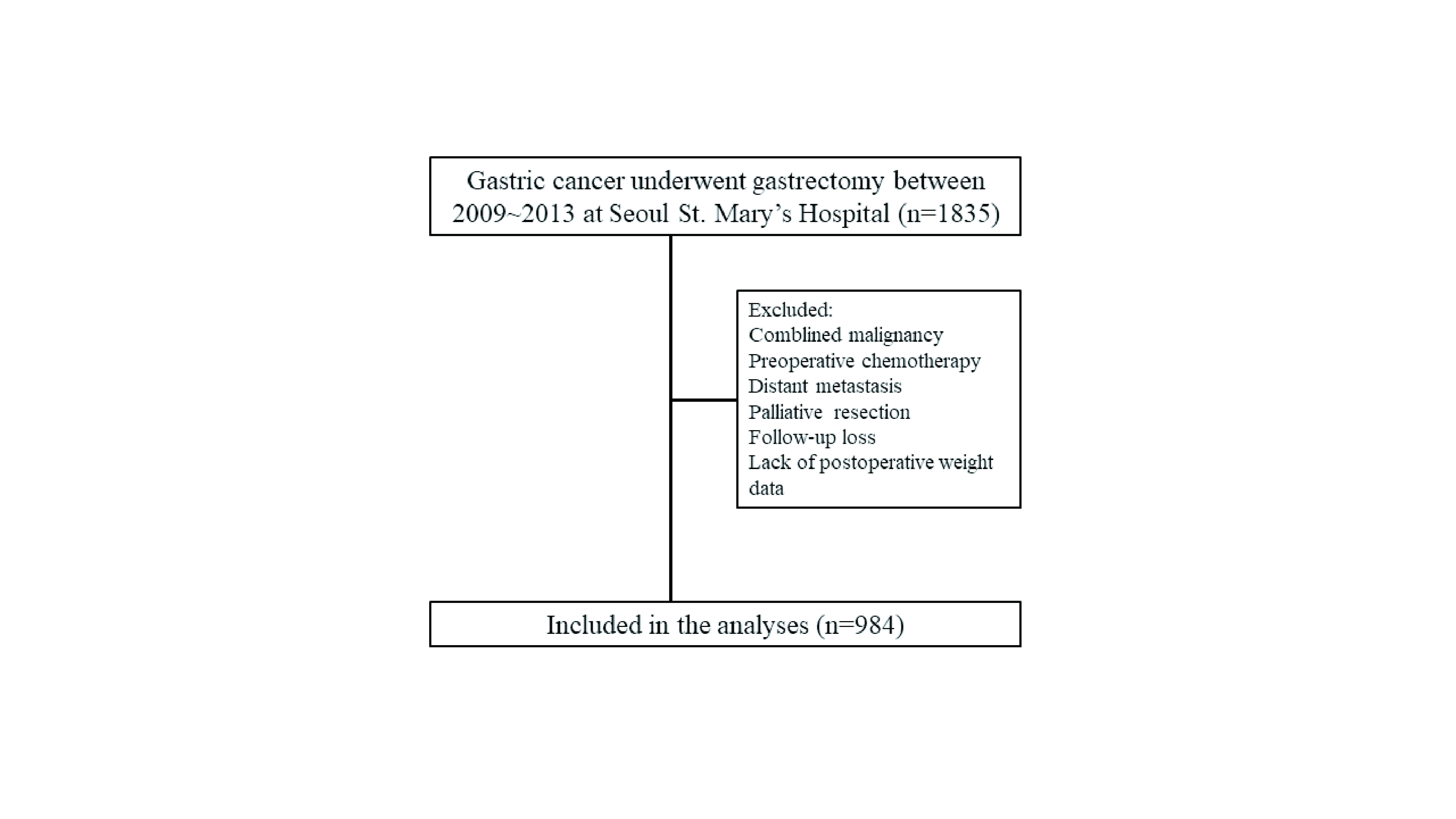

Supplement: Supplementary file 1 — Additional file 1: Supplementary Fig. 1. Flow chart of the study cohort with the inclusion and exclusion criteria. [file 12885_2023_11050_MOESM1_ESM.jpg]
