# Supplementary material for: Predictive model for long-term weight recovery after gastrectomy for gastric cancer: an introduction to a web calculator
Source: BMC Cancer. 2023 Jun 23;23:580. doi: 10.1186/s12885-023-11050-7 (PMC10288751; doi:10.1186/s12885-023-11050-7)
Supplement: Supplementary file 2 — Additional file 2: Supplementary Table 1. Demographic and clinicopathological variables. Supplementary Table 2. Characteristics of the subgroups by the trends in body weight change after gastrectomy. [file 12885_2023_11050_MOESM2_ESM.docx]

**Supplementary Table 1** Demographic and clinicopathological variables

| **Variables, n(%)** | **All patients (N = 984, 100%)** |
| --- | --- |
| **Age (years)** |  |
| < 65 | 706 (71.7%) |
| ≥ 65 | 278 (28.3%) |
| **Sex** |  |
| Male | 652 (66.3%) |
| Female | 332 (33.7%) |
| **ECOG** |  |
| 0 | 736 (74.8%) |
| 1 | 233 (23.7%) |
| ≥ 2 | 15 (1.5%) |
| **Preoperative BMI (kg/m^2^)** |  |
| < 23 | 369 (37.5%) |
| ≥ 23 | 615 (62.5%) |
| **Resection** |  |
| STG | 742 (75.4%) |
| TG | 242 (24.6%) |
| **pTNM stage** |  |
| Ⅰ | 775 (78.8%) |
| Ⅱ | 139 (14.1%) |
| Ⅲ | 70 (7.1%) |

*Abbreviations*: *ECOG* Eastern Cooperative Oncology Group performance status, *BMI* body mass index, *STG* subtotal gastrectomy, *TG* total gastrectomy

**Supplementary Table 2** Characteristics of the subgroups by the trends in body weight change after gastrectomy

| **Variables, n(%)** | **G1 (N = 13, 100%)** | **G2 (N = 554, 100%)** | **G3 (N = 375, 100%)** | **G4 (N = 42, 100%)** | **p-value** |
| --- | --- | --- | --- | --- | --- |
| **Age (years)** |  |  |  |  | **< 0.001** |
| < 65 | 11 (84.6%) | 425 (76.7%) | 246 (65.6%) | 24 (57.1%) |  |
| ≥ 65 | 2 (15.4%) | 129 (23.3%) | 129 (34.4%) | 18 (42.9%) |  |
|  |  |  |  |  |  |
| **Sex** |  |  |  |  | **0.018** |
| Male | 7 (53.8%) | 346 (62.5%) | 268 (71.5%) | 31 (73.8%) |  |
| Female | 6 (46.2%) | 208 (37.5%) | 107 (28.5%) | 11 (26.2%) |  |
|  |  |  |  |  |  |
| **ECOG** |  |  |  |  | 0.139 |
| 0–1 | 13 (100.0%) | 549 (99.1%) | 367 (97.9%) | 40 (95.2%) |  |
| ≥ 2 |  | 5 (0.9%) | 8 (2.1%) | 2 (4.8%) |  |
|  |  |  |  |  |  |
| **Preoperative BMI (kg/m^2^)** |  |  |  |  | **< 0.001** |
| < 23 | 10 (76.9%) | 203 (36.6%) | 122 (32.5%) | 34 (81.0%) |  |
| ≥ 23 | 3 (23.1%) | 351 (63.4%) | 253 (67.5%) | 8 (19.0%) |  |
|  |  |  |  |  |  |
| **Resection** |  |  |  |  | 0.262 |
| STG | 10 (76.9%) | 417 (75.3%) | 278 (74.1%) | 37 (88.1%) |  |
| TG | 3 (23.1%) | 137 (24.7%) | 97 (25.9%) | 5 (11.9%) |  |
|  |  |  |  |  |  |
| **pTNM stage** |  |  |  |  | **0.003** |
| EGC | 10 (76.9%) | 425 (76.7%) | 314 (83.7%) | 26 (61.9%) |  |
| AGC | 3 (23.1%) | 129 (23.3%) | 61 (16.3%) | 16 (38.1%) |  |
|  |  |  |  |  |  |
| **Postoperative weight change to 1year** |  |  |  |  | **< 0.001** |
| (-) ↓ |  | 554 (100.0%) | 375 (100.0%) |  |  |
| (+) ↑ | 13 (100.0%) |  |  | 42 (100.0%) |  |
|  |  |  |  |  |  |
| **Hemoglobin change** |  |  |  |  | **< 0.001** |
| (-) ↓ | 3 (23.1%) | 92 (16.6%) | 90 (24.0%) | 17 (40.5%) |  |
| (+) ↑ | 10 (76.9%) | 462 (83.4%) | 285 (76.0%) | 25 (59.5%) |  |
|  |  |  |  |  |  |
| **Albumin change** |  |  |  |  | **0.009** |
| (-) ↓ | 1 (7.7%) | 178 (32.1%) | 133 (35.5%) | 22 (52.4%) |  |
| (+) ↑ | 12 (92.3%) | 376 (67.9%) | 242 (64.5%) | 20 (47.6%) |  |
|  |  |  |  |  |  |
| **TIBC class** |  |  |  |  | 0.417 |
| Low/Normal | 12 (92.3%) | 536 (96.8%) | 364 (97.1%) | 39 (92.9%) |  |
| High | 1 (7.7%) | 18 (3.2%) | 11 (2.9%) | 3 (7.1%) |  |
|  |  |  |  |  |  |
| **Ferritin class** |  |  |  |  | 0.775 |
| High |  | 6 (1.1%) | 3 (0.8%) | 1 (2.4%) |  |
| Low/Normal | 13 (100.0%) | 548 (98.9%) | 372 (99.2%) | 41 (97.6%) |  |

*Abbreviations*: *ECOG* Eastern Cooperative Oncology Group performance status, *BMI* body mass index, *STG* subtotal gastrectomy, *TG* total gastrectomy, *EGC* early gastric cancer, *AGC* advanced gastric cancer, *TIBC* total iron-binding capacity
